# The ethics and safety of medical student global health electives

**DOI:** 10.5116/ijme.5334.8051

**Published:** 2014-04-10

**Authors:** Evelyn M. Dell, Lara Varpio, Andrew Petrosoniak, Amy Gajaria, Anne E. McCarthy

**Affiliations:** 1 Department of Medicine, Emergency Medicine, University of Toronto, Toronto, Canada; 2 Department of Medicine, University of Ottawa, Academy for Innovation of Medical Education, Canada; 3 Department of Medicine, Psychiatry, University of Toronto, Canada; 4 Department of Medicine, Infectious Disease/Global Health, University of Ottawa, Canada

**Keywords:** Ethics, international medical education, medicine, clinical

## Abstract

**Objectives:**

To explore and characterize the ethical and safety challenges of global health experiences as they affect medical students in order to better prepare trainees to face them.

**Methods:**

Semi-structured interviews were conducted with 23 Canadian medical trainees who had participated in global health experiences during medical school. Convenience and snowball sampling were utilized. Using Moustakas’s transcendental phenomenological approach, participant descriptions of ethical dilemmas and patient/trainee safety problems were analyzed. This generated an aggregate that illustrates the essential meanings of global health experience ethical and safety issues faced.

**Results:**

We interviewed 23 participants who had completed 38 electives (71%, n=27, during pre-clinical years) spend-ing a mean 6.9 weeks abroad, and having visited 23 countries. Sixty percent (n=23) had pre-departure training while 36% (n=14) had post-experience debriefing. Three macro-level themes were identified: resource disparities and provision of care; navigating clinical ethical dilemmas; and threats to trainee safety.

**Conclusions:**

Medical schools have a responsibility to ensure ethical and safe global health experiences. However, our findings suggest that medical students are often poorly prepared for the ethical and safety dilemmas they encounter during these electives. Medical students require intensive pre-departure training that will prepare them emotionally to deal with these dilemmas. Such training should include discussions of how to comply with clinical limitations.

## Introduction

The genesis of this study dates back to 2007, when our research team launched a qualitative needs assessment to inform the development of educational materials for trainees embarking on global health experiences (GHEs).^1^^,^^2^ Part of that study’s data collection included interviews using broad-scoped, open-ended questions (e.g. “Can you tell me a particularly memorable story about your GHE?”) with medical trainees who had previous clinical global health exposure. In response, participants repeatedly and emotionally recounted incidents of ethical and safety-related dilemmas, including risks of needle sticks, doing clinical work that exceeded skill levels, and being publically ridiculed by host-country physician mentors. We felt that the theme of trainees encountering significant ethical dilemmas identified by our needs assessment data warranted further investigation. Those insights prompted the development of a second study, described here.Increasingly, medical students in both pre-clinical and clinical years are travelling from high to low-resource settings for clinical electives^3^ where they may face complex ethical challenges.^4^^,^^5^ Though often not well characterized, these challenges have included students having minimal clinical impact with the sense of being a burden rather than an aid to local communities,^4^^-^^9^ students acting beyond their scope of practice under minimal supervision,^10^^-^^12^ working with limited clinical resources,^4^^,^^8^ and experiencing communication dilemmas.^5^^,^^9^^,^^11^ In addition, while GHE trainee safety concerns are frequently cited,^7^^,^^13^^,^^14^ little elaboration is made regarding the specific nature of risk with the exception of HIV risks.^4^^,^^15^^-^^17^ Other important safety issues in GHEs that deserve attention yet remain inadequately studied include motor vehicle crashes, non-HIV infectious diseases, drowning, gun and knife violence, sexual assault, and mental health issues.^7^^,^^16^^,^^18^Recent publications warn of the potential harms of GHEs that span complex ethical and professional issues,^6^^,^^8^^,^^9^^,^^19^ including threats to patient and trainee safety,^13^^,^^15^ difficulty providing culturally-sensitive care,^13^ and trainees performing procedures in under-supervised settings.^5^^,^^20^ Yet, despite these challenges, the proliferation of GHEs continues owing largely to the well-documented trainee benefits associated with GHEs, including increased preferences to work with underserved populations and a greater likelihood of selecting a career in primary care.^17^^,^^18^^,^^21^^-^^25^Current medical literature suggests that the potential harms faced through GHE participation may be reduced with improved pre-departure training (PDT), well-defined objectives, and better consideration for the needs of host communities.^2^ In both Canada^26^ and the United States,^27^ national guidelines and information resources concerning GHEs are being, or have been developed. Additionally, issues related to the ethics of GHE have recently been addressed by an advisory group to the World Health Organization.^7^ Despite these efforts, considerable variability subsists between medical schools in trainee requirements for participation in GHEs.^28^^-^^31^Two important knowledge gaps were identified in the literature–the sparse characterization of commonly encountered ethical scenarios and data related to non-HIV safety issues during multi-week GHEs. Recent medical student interviews^5^^,^^6^ offered some insight into such ethical scenarios; however, trainee-specific safety concerns received little attention. Overall, very few studies have specifically investigated the lived experience of medical trainees during GHEs. Consequently, details of how students experience GHEs are not well understood. Given the growing number of students participating in GHEs^32^^,^^33^ research must scrutinize GHE-related ethical and safety dilemmas in order to responsibly advocate medical trainee participation. We conducted a qualitative study hinged on two specific research questions:

1. What is the nature of medical students’ experiences of ethical and safety-related challenges during GHEs?
2. How (in what contexts) did they experience these challenges?

Our objectives were to explore and characterize the complexity of ethical and safety-related dilemmas faced by medical students during GHEs. We aim to inform the training process of GHEs in order to better equip trainees to face such dilemmas.

## Methods

### Study design

This study received approval from the University of Ottawa Research Ethics Board. Informed consent was collected from all participants. We conducted a phenomenological study to identify commonalities across medical student (i.e. within first 4 years of undergraduate medical training) experiences of ethical and safety-related challenges faced during GHEs. We selected phenomenology since this approach to qualitative inquiry aims at “reduc[ing] individual experiences with a phenomenon to a description of the universal essence.”^34^ More specifically, this study drew on the transcendental phenomenological tradition as employed by Moustakas.^35^Phenomenology has strong roots in specific philosophies and it is argued that a study employing this qualitative approach would be remiss in not explaining its philosophical presuppositions.^34^ Therefore, we briefly describe our phenomenological approach in this methods description, but include details regarding transcendental phenomenology and Moustakas’s transcendental approach in Appendix 1.Phenomenology seeks to understand what it means to be in the world, or to have a particular life experience, without imposing previous assumptions about what is “real” about those experiences.^36^ Each school of phenomenology has its own particular view about what constitutes a world view and the nature of being human in that world.^37^ It is important to note that each approach to phenomenology represents a philosophy that studies lived experiences in different ways.^38^ While many approaches to phenomenology exist, this study draws on Husserl’s transcendental phenomenology.

### Participants

We recruited 23 medical trainees within a Canadian academic medical centre (see demographic description of participants in Table 1). All participants had engaged in GHEs in resource-limited settings during their undergraduate medical training.

**Table 1:** Participant demographics

Demographics	
Total number of trainees interviewed	23
Gender of trainee interviewees	
Male	9
Female	14
Total number of global health experiences (GHE)	38
Average number of weeks per trainee spent on GHEs	6.85
Level of training during GHE	
Medical student year 1	47%
Medical student year 2	24%
Medical student year 3	8%
Medical student year 4	21%
Continents visited	
Africa	53%
Americas	21%
Asia	16%
Other	11%
Trainees who participated in pre-departure training	60%
Trainees who participated Debriefing	36%
Average number of years between most recent GHE and interview	1.47

### Sampling and sample size

Interviewees were selected from a convenience sample of 42 individuals known to the researchers, with snowball sampling to identify additional participants. Demographic data was collected using a brief survey prior to each interview.Our team was prepared to approach and, if applicable, interview up to 40 students in search of thematic saturation. In designing the study, we assumed that not all available participant recruits would meet our single inclusion criteria (i.e. participants must have personally faced or have witnessed an ethical- or safety-related challenge during a GHE undertaken during undergraduate medical training). We were surprised when every participant we approached met this criterion. We interviewed participants until thematic saturation was reached (n=23).

### Data collection

Data were collected through one-on-one, semi-structured interviews. The first iteration of our interview protocol consisted of only three open-ended questions (see Appendix 2). Protocol questions were modified during data collection, in an iterative fashion, to fully vet emerging themes (see Appendix 3 for the final interview protocol). Interviews lasted approximately 30-45 minutes. A trained qualitative researcher conducted and audio recorded each interview. Interviews were anonymized during transcription. Gender and year of medical training were the only participant-identifying features retained (F, M = female or male, respectively; MS#, PGY# = year of medical school or residency training at time of interview, respectively).

### Procedure and data analysis

#### Moustakas’ approach to transcendental phenomenology

Following Moustakas’ systematic analysis steps,^35^ our team (1) described our own experiences with the phenomenon (epoche); (2) read the data transcripts to isolate meaningful statements concerning the phenomenon (horizontalization); (3) organized these utterances into thematic clusters (meaning units); (4) integrated these themes into descriptions of participant experiences (textual and structural descriptions); and (5) produced a merged account of the “essences” of participant meanings (intuitive integration). Across all steps, analytical memos, meeting minutes, and coding structure revisions were documented to ensure confirmability.^43^ Data were coded using NVivo software to facilitate cross referencing (Version 8, QSR International).

#### Epoche

We undertook epoche to set aside subjective perspectives so that our analysis would be as unbiased as possible. The description of our epoche begins with the history of this study as described in the introduction. To this we add the following description of our individual GHE experiences and academic training. E.D, L.V, and A.M. are from the University of Ottawa. During active research, E.D. was a senior medical student. She has participated in several GHEs and completed a Masters in Public Health with thesis project in Madagascar. L.V. is an Assistant Professor level qualitative researcher in medical education with global health research experience, but no direct GHE experience. A.M. is Director of the Office of Global Health at the Faculty of Medicine, with 20 years of experience participating in and preparing trainees for global health experiences. A.G and A.P are at the University of Toronto. A.P. is an emergency medicine resident who completed GHEs in Vietnam and Botswana and who has experience in global health education research. A.G. is a resident in Psychiatry who completed GHEs in India, Brazil, and Laos and has conducted research on global mental health and pediatric mental health.Aware of each other’s experiences and training, we explored our biases, presuppositions and cultural expectations. The diversity of our team required regular reflections on the data (i.e. analysis meetings regularly brought attention to the “actual words in the transcripts”).

#### Horizontalization

We identified over 750 participant utterances that provided information about medical trainee experiences with ethical- and safety-related challenges experienced during GHEs.

#### Meaning units

Utterances that were deemed irrelevant to the research questions and that were considered one-off (topics addressed by only one participant) tangents were removed. Overlapping and repeated statements were examined to generate thematic statements of shared related topics/concerns, resulting in macro-level meaning units (themes). From these meaning units, our research team then created a textual description (what was experienced) and a structural description (how it was experienced). It was only at this stage in the analysis process that different perspectives, including those from existing research literature, were considered as a means for shaping the meaning unit descriptions. This analysis is detailed in the discussion.

#### Intuitive integration

Finally, textual and structural descriptions were synthesized into a composite description, or the “essence,” of the ethical and safety-related dilemmas faced by medical students during GHEs. This is described in the final paragraph of the paper.

## Results

The study’s 23 participants completed a total of 38 GHEs (Table 1). Eighty three percent 83% (n=19) of participants described witnessing or being involved in scenarios where GHE-participant physical safety was jeopardized. These scenarios included occupational safety concerns (60.9%, n=14), including risk of infectious disease exposure from potential needle stick injuries (21.7%, n=5), as well as life-threatening scenarios outside the clinical setting (52.2%, n=12). Examples of such scenarios included road safety issues and the nearby murder of Western tourists. In addition, 57% (n=13) of participants described being involved in and/or witnessing an ethical dilemma.Three macro-level meaning units were developed during analysis: resource disparity, navigating ethical dilemmas, and trainee health and safety. Two dominant subthemes are described for each macro-level meaning unit. Select interview quotes illustrate these subthemes. Participants are identified by interview numbers and, when possible, identified by the level of training achieved at the time of their interview.

### Resource disparity

#### Limited medical supplies impacting on patient care

Trainees expressed having ethical concerns about local standards of care observed during GHEs. They described a lack of affordable medical supplies affecting patient safety. As one first-year GHE participant explained:

“Patient care was very compromised. A lot of patients really needed tests or blood transfusions and could not afford them and therefore they were just left to just bleed to death.” *(03 – F, MS4)*

Similarly, following an obstetrics elective, another first-year student contrasted patient care in Canada to that in East Africa, describing lack of affordable resources as a barrier to implementing best practices in the global health context:

“ [It is] not best practice [compared to] certain interventions we do in Canada…There was a woman that had an incomplete abortion and she still had products of conception that were in her uterus…[In Canada] we would anesthetize the patient and do one [evacuation] under anesthetic. Whereas over there, they use no anesthetic because they just don’t have the resources or the money to pay for anesthetic unless the patient pays, which no one can.” *(02 – M, MS4)*

#### Providing unsupervised care beyond scope of training

Patient care was perceived as negatively impacted by medical personnel shortages. Without enough local physicians, trainees were faced with managing sick patients independently, beyond their level of training. One student described the scenario of either treating patients without adequate knowledge or letting them suffer and die:

“A lot of times I was put in situations where there was somebody bleeding in front of me, and I really didn’t know what to do. So I would just do what I could, and hope [I was] doing the right thing. A lot of times there was just nobody to help you and so you are all by yourself” *(03 – F, MS4)*

Another trainee discussed feeling obliged to complete clinical activities beyond her skill level after a local staff physician quit. She was left without adequate clinical supervision, but also left in an ethical dilemma vis a vis patient safety:

“You could probably give a different definition to patient safety in a place where people don’t even have access to services whatsoever. Patient safety was routinely compromised…There was an expectation that when you come to volunteer all the way from Canada, you should be able to act as a physician or in a major head role because they’re so under-served…And that’s when you can get in over your head.” *(12 – F, MS4)*

### Navigating ethical dilemmas

#### Communicating skill level with host supervisor to avoid patient harm

As trainees were faced with decisions about engaging in clinical care beyond their skill level, many felt compelled to advise their host supervisors of their limited abilities. These discussions occurred so as to avoid performing procedures that could lead to patient harm. However, limited skills were not always viewed by local supervisors as a reason to avoid performing procedures, requiring trainees to persist in declining such opportunities:

“They would have been fine with me doing a bunch of things. The nurses kept pushing [me] to take blood and stuff but I didn’t feel comfortable with it…They didn’t have gloves [or] very sterile techniques [pause] and I also just wasn’t comfortable learning those techniques on children…They just kept saying to me ‘You can try, you can try. It’s no big deal, you haven’t done it.’ And so I kept saying no.” *(07 – M, MS2)*

Some local supervisors’ misconceptions of the skills of “western-trained medical students” persisted despite these discussions. Eventually ethically challenging situations occurred where trainees felt ridiculed for their limited skill even when asked to do a procedure clearly beyond their skill level:

“Residents would ask: ‘why don’t you do that thoracotomy?’ ‘Well I am a first year med student. I don’t know how [so] I am not going to do it.’…A lot of times the residents or staff would laugh at you for not knowing how to do certain procedures. It was embarrassing.” *(03 – F, MS4)*

#### Having minimal clinical impact

While trainees often declined clinical opportunities beyond their skill level, they did engage in less demanding patient care, and acknowledged their limited clinical impact during GHEs. Frequently, trainees felt they benefited more from GHEs than host communities. As first year student GHE participant recalled:

“The main ethical issue I had was my own feelings of [being] a medical tourist…I had a lot of trouble with that...Although I took money and donated it to the hospitals and I did try and contribute, I basically told myself I would never go back to a developing country until I had something to offer, I felt like it was very one-sided, that all I did was take, take, take.” *(23 – F, PGY1)*

One interviewee acknowledged the limited contribution of trainees with only pre-clinical experience. He admitted his contribution was likely negligible:

“For some reason we thought that we could go over there and [help] these people from a medical point of view but we only had classroom learning…So when people ask about my experience I always try to discourage [medical students] from doing observerships because you can’t really contribute.” *(19 – M, PGY1)*

### Trainee health and safety

#### Impacting trainee psychological wellbeing

Trainees felt that GHEs presented ethical and psychological challenges, often as a consequence of resource disparity. Trainees primarily experienced guilt when unable to provide care to everyone. For some trainees, these challenges led to signs of poor long-term coping, often exacerbated by poor supervision and inadequate numbers of local staff. One trainee described her emotional strife after performing a lumbar puncture (LP) for the first time without proper instruction or supervision:

“You feel bad for the patient…Here we are [myself and a local trainee] learning how to do an LP on [a patient] and this is both of our first time. There is no staff to teach you…You get into a very sticky situation. It is either me doing it for the first time or that resident. You felt terrible as a human being [thinking] ‘Am I really going to help this person? No. Most likely they will die from an infection because I am doing something wrong but there is nobody to teach me.’ ” *(03 – F, MS4)*

Another first-year student similarly described guilt along with difficulty coping with the poverty and poor patient outcomes encountered. She summarized her emotional response to an encounter with a malnourished baby who died, in part, because the mother could not afford food and healthcare:

“Honestly it was probably one of the most traumatic things to have happened to me and it took a very long time [pause] after we came back [to Canada] to be able to even go through the motions…When it happened, I crashed. I got home in the afternoon and stayed in bed until the morning and I didn’t speak to anyone. It was ridiculously hard on me.” *(09 – F, MS2)*

#### Threatening physical safety

Trainees also described threats to their physical wellbeing during GHEs. Specifically, threats were related to occupational safety, local political and cultural climate, and risk of transmittable diseases. One participant described a traumatic experience of a personally experienced sexual assault, one that she attributed to her lack of cultural knowledge and overall preparedness:

“I was less prepared for some of the cultural [and] social differences and how to protect myself…So I was kind of stuck in this car with a drunk man who was trying to do things that he shouldn’t have been doing and I didn’t know how to get out. I didn’t know what to do.” *(03 – F, MS4)*

A first-year student discussed her poor understanding of the local political environment during a GHE. She was not aware of potential dangers, until two violent incidents occurred during her elective:

“There were a lot of political things that we didn’t fully understand… [At] a neighboring hostel, while we were there, two tourists were murdered. That was really scary. And then a second incident happened, a tourist was kidnapped and beheaded.” *(16 – F, PGY1)*

## Discussion

This study confirms that medical trainees participating in GHEs are regularly placed in ethical- and safety-related dilemmas. The three main macro-level meaning units reported describe, what is the nature of medical students’ experiences of ethical and safety-related challenges during GHEs, and their subthemes categorize how they experienced these challenges (see Table 2). Despite the fact that 60% of our interviewees participated in some form of pre-departure training, they experienced strong feelings of frustration, anxiety, and even emotional trauma as a result of encountering these dilemmas. In other words, for 60% of our participants, their pre-departure training did not adequately prepare them for the ethical and safety dilemmas they faced. While this could be interpreted as a comment on the associated pre-departure training programs, we suggest that these findings are indicative of a more pervasive problem. As the following discussion illustrates, many studies report that the majority of trainee GHE participants experience similar challenges with respect to resource disparity, navigating ethical dilemmas, and trainee health and safety (i.e. our “what” description). We suggest that descriptions of how these macro-level themes are experienced, descriptions provided in this study, can provide useful insights how we might address these shared challenges.

**Table 2:** The “what” and “how” descriptions of medical student experiences of ethical- and safety-related challenges faced during GHEs

What is the nature of medical students’ experiences of ethical- and safety-related challenges faced during GHEs	How (in what contexts) did they experience these challenges
Resource disparity	1. Limited medical supplies impacting on patient care
	2. Providing unsupervised care beyond scope of training
Navigating ethical dilemmas	1. Communicating skill level with host supervisor to avoid patient impact
	2. Having minimal clinical impact
Trainee health and safety	1. Impacting trainee psychological wellbeing
	2. Threatening physical safety

Other research has reported that trainees are often shocked with local resource disparities, feeling that unaffordable medical supplies^44^^,^^45^ and low doctor-to-patient ratios^46^^,^^47^ strongly influenced health outcomes. Also, a recent publication describes several potential harms of short term GHEs during one-week electives by medical students. These authors noted concerns among participants regarding lack of supervision and inadequate clinical skills.^6^ Trainees who travel to low resource GHE settings often describe resource disparity as a common denominator to feeling like a medical tourist.^2^

We propose that pre-departure training should strive to better equip trainees (a) to work with the limited resources that will be available at hosting institutions and (b) to work within clinical environments where supervision may be limited but patient needs will be high. As one of our participant quotations illustrates, students participating in GHEs can find themselves clinically “in over their heads” and without the tools they usually rely on to stay afloat. Pre-departure training that focuses on these aspects of how resource disparity will be experienced could help trainees avoid feeling that they were left holding the proverbial “bag.” These experiences of resource disparity may have repercussions that extend beyond the GHE experience. We cannot assume that their long-term impact is moot. In addition, other dilemmas such as congruence for expectations and procedural competency during GHEs have not been well studied, though several studies allude to such issues,^20^^,^^48^ future studies should explore the frequency and potential impact of procedures completed by untrained/unsupervised students.During GHEs, our data suggests that there were considerable communication barriers that prevented trainees from adequately explaining their level of training. Indeed, trainees were frequently involved in ethically compromising situations when local supervisors misunderstood trainee skill.Other research has highlighted that the need to navigate ethical dilemmas is not isolated to medical trainees during GHEs.^49^ Trainees in a North American context have acknowledged that they receive greater benefit from the trainee-patient interaction than patients, and they have expressed concern that their inexperience may lead to patient harm.^50^ This echoes our finding that medical students who participate in GHEs feel that they have minimal clinical impact. As the cited participant remarks highlight, participating in GHEs as a medical student is an exercise in “observership.” However, the experiences for medical students during their North American clinical experiences differ in the level of supervision and established educational objectives. During a GHE, the supervisor, if present, may not understand the clinical experience of a pre-clinical medical student. In addition, educational objectives may not be adequately addressed especially when time or personnel are limited. So while trainees may express concern that they benefit more than the patient during patient interactions, additional challenges place trainees at risk for ethical dilemmas during GHEs. In our study, approximately 70% of participants participated in pre-clerkship GHEs with minimal clinical exposure prior to departure. The predilection of less experienced trainees participating in GHEs appears generalizable. Thirty percent (30%) of North American medical students complete GHEs by the time of graduation,^33^ while 10%^51^ and 14%^42^ of Pediatrics and General Surgery residents, respectively, have done so. Assuming this trend continues, reported experiences of minimal clinical impact will likely increase.While the need to navigate such ethical dilemmas in GHEs will persist, pre-departure training programs could focus on *how* these dilemmas are experienced and aim to arm trainees to engage in the ethically challenging contexts of GHEs. For example, pre-departure training could provide students with strategies through case-based learning to relay their skill and comfort levels to preceptors before clinical activities begin and during care. In our study, most trainees described discussions ranging from awkward to humiliating when they declined procedures to preserve patient safety. Trainees need to be prepared to have these conversations. Additionally, the anxiety of having minimal clinical impact is another ethical dilemma that we can prepare trainees to face.Also, it is important to ensure that GHEs are run in collaboration with local communities so as to improve the experience for the participants, local patients and receiving institutions. The local host perspectives are essential and must inform GHE design and implementation. A previous study in the Solomon Islands found that the majority of health care providers believe medical students can diagnose, perform procedures, and prescribe medications independently,^10^ while another study in Guatemala found host perceptions to be highly variable.^53^ This demonstrates the need for additional studies to uncover and clarify how host communities perceive and experience GHEs.Additional guidelines outlining clear learning objectives among pre-clinical and clinical level trainees during GHEs could also help to prepare trainees. The ethical dilemmas that occur particularly among less experienced trainees highlight the value of institutional partnerships between the sending and receiving sites. In our study, none of the trainees participated in GHEs that were part of formal established programs between institutions. Future research should evaluate the utility of formalized institutional partnerships and whether they improve patient and trainee-oriented outcomes with reductions in harm.While our data is insufficient to make recommendations regarding the appropriateness of GHEs for all medical students, our findings suggest that this question of appropriate minimum training level for GHE participation is important to consider. A similar consideration is echoed in recent research that asks if the minimum global health competencies required for GHEs is beyond that achievable by pre-departure training alone. As argued by Huish, the social justice and ethics of global health are deep and complex, and related competencies are likely more thoroughly developed if embedded within the medical curriculum or even as a pre-requisite course prior to entering medical school.^54^Finally, and consistent with previous studies,^14^^-^^16^ our data suggests trainees commonly experienced threats to physical and emotional wellbeing. Our findings highlight the serious threats faced by trainees during GHEs, and the clear psychological impact that these events have on trainees. Understanding how GHE-related health and safety concerns impact trainee wellbeing is critical for successful and continued GHE implementation. Approaches for securing personal safety in these contexts must be provided prior to the elective. This could include tertiary prevention with an immediate plan of action (e.g. who to contact and how) should any critical incident occur. Additionally, as the participant descriptions provided here illustrate, the guilt, and both emotional and physical trauma that can result from GHE participation are keenly felt. These situations demonstrate the importance of formal post-elective debriefing to ensure on-going student wellbeing.We acknowledge that our study has limitations. The interviews were conducted at a single Canadian Medical School by individuals who self-selected to be interviewed; however the heterogeneity of experiences, sponsoring agencies and geographic location, as well as previous publications, suggest that the results apply to a broader group of medical trainees. There is also concern about recall bias, as some interviews occurred a few years after GHEs. As well, while GHEs took place in a variety of continents, over 50% were in Africa, which could have biased the prevalence of certain themes in our data set. Future research should involve data collection from all GHE trainee participants both before and after their experiences, including participants from several different medical schools who travel to a wide range of GHE locations. We also suggest that future studies investigating the ethical and safety dilemmas of GHEs should collect detailed information about the GHE location to support thematic cross referencing. These details would include delineating experiences in rural versus urban areas; private versus public clinics; and in-country GHE participant support structures. We suggest these details could help inform location-specific PDT curricula.

## Conclusion

In conclusion, trainees experience complex ethical dilemmas and safety challenges during GHEs that fall under three macro-level themes: (1) resource disparity impacting care, (2) navigation of ethical dilemmas, and (3) trainee health and safety. Our findings suggest that ethical and safety issues are not adequately addressed in training surrounding GHEs and that trainees are not prepared to handle these challenges. Also, we submit that GHE participants will greatly benefit from formal debriefing sessions. As more undergraduate trainees participate in GHEs, it is important that GHEs abide by medicine’s fundamental rule of primum non nocere. The well-documented benefits of GHEs must never be outweighed by their harms.

## 

### Conflict of Interest

The authors declare that they have no conflict of interest.

## Appendix 1.

### Transcendental phenomenology

Husserl’s philosophies about science gave rise to transcendental phenomenology. A key premise was that experience, as it was perceived by an individual’s consciousness, had scientific value and was worthy of rigorous examination.^39^ Husserl’s transcendental phenomenology promised to disclose a realm of being that arose from experience and that, by probing deeper and deeper into this reality, could reach true essential meaning.^40^ Descriptions of essential meanings allowed researchers to build knowledge of reality. Central to this is the researcher’s bracketing off of prior personal knowledge, attitudes, beliefs and prejudices in order to grasp essences.^36^ The emphasis of transcendental phenomenology is on description of these essential meanings, rather than the researcher’s interpretations of the descriptions.

### Why transcendental phenomenology

We chose to employ transcendental phenomenology to understand the essence of trainee experiences with ethical- and safety-related challenges when engaged in GHEs. While a hermeneutic approach could have generated important insights, hermeneutic phenomenology requires a historically situated and contextually informed interpretation of data to gain meaningful understanding.^35^ We felt that it was not possible to examine and interpret participant experiences in their historically situated and contextually informed richness given the heterogeneous character of the GHEs (e.g. diverse in relation to location, host expectations, trainee expectations, cultural contexts, in-country support, etc.). There were simply too many unique aspects of each GHE for our team to hermeneutically generate interpretations. Instead, we employ transcendental phenomenology to describe the essence of the ethical- and safety-related challenges faced by undergraduate medical students during GHEs.

### Moustakas’ approach to transcendental phenomenology

Moustakas’ approach to transcendental phenomenology is hailed as a useful approach when there is (1) an identified phenomenon to understand, and (2) individuals who can describe their experiences with that phenomenon.^41^ This was precisely the context of this study. Our previous work had identified a phenomenon (i.e. ethics- and safety-related challenges faced by medical students during GHEs) and we had ready access to participants who had experiences with this phenomenon (i.e. medical trainees in our institution who had participated in GHEs). Also, Moustakas provides a structured approach for the steps of phenomenological analysis. Finally, Moustakas’ detailed analysis steps can be confirmed through review of audit trails, thus improving the transferability of study findings.

## Appendix 2.

### Interview protocol – first iteration

1) Tell me a little about your previous global health experiences (GHEs). (Icebreaker)

a. Where and when was the GHE?

b. How was it organized?

c. How long was it?

d. Was it more clinical / hands on or more of an observership?

2) During your GHEs, were there any occasions in which you or other trainees were involved in situations where you were concerned about patient safety?

Prompt: If participant replies positively / “yes”, ask: Can you give me an example or a story about such an experience?

3) While you were abroad, were there any clinical situations where you were concerned about your own or another trainee’s personal safety?

Prompt: If participant replies positively / “yes”, ask: Can you describe that situation for me?

4) During your GHEs, were there any occasions in which you or other trainees were involved in situations where you or another trainee were placed in an ethical dilemma?

Prompt: If participant replies positively / “yes”, ask: Can you give me an example or a story about such an experience?

## Appendix 3.

### Interview protocol – final iteration

Exploring the ethics and safety of global health experiential learning: addressing participant and host communities

Question List

1) Tell me a little about your previous global health experiences (GHEs). (Icebreaker)

a. Where and when was the GHE?

b. How was it organized?

c. How long was it?

d. Was it more clinical / hands on or more of an observership?

2) During your GHEs, were there any occasions in which you or other trainees were involved in situations where you were concerned about patient safety?

Prompt: If participant replies positively / “yes”, ask: Can you give me an example or a story about such an experience?

3) While you were abroad, were there any clinical situations where you were concerned about your own or another

trainee’s personal safety?

Prompt: If participant replies positively / “yes”, ask: Can you describe that situation for me?

4) During your GHEs, were there any instances where you or another trainee were asked or felt pressured to perform above your clinical competency or skill level?

Prompt: If participant replies positively / “yes”, ask: Can you give an example or a story?

5) While you were abroad, were there any clinical situations that you would describe as ethically questionable either

involving you or other trainees?

Prompt: If participant replies positively / “yes”, ask: Can you tell me about that situation

6) Were there any situations where you were uncomfortable with the way a healthcare provider was working with a

patient?

Prompt: If participant replies positively / “yes”, ask: Can you tell me about that situation?

7) Did you have any supervision or support of any kind while you were completing the GHE?

a. Please specify (how often, who were they, what their role was etc.)

b. Would you change anything?

8) Did you ever complete any pre-departure training and / or debriefing?

a. Why / why not?

b. Was it / would it have been helpful?

c. Who was it provided by / how long was it?

d. If it had been offered would you have done it? Why/Why not?

Great, thank you very much. Do you have any questions for me at this point? - Thank you again for your participation.
